# Recurrent Oral Mucocele Management with Diode Laser

**DOI:** 10.1155/2020/8855759

**Published:** 2020-10-03

**Authors:** Amira Besbes, Yamina Elelmi, Faten Khanfir, Raja Belgacem, Hichem Ghedira

**Affiliations:** ^1^University of Monastir, Faculty of Dental Medicine, 5019 Monastir, Tunisia; ^2^University Dental Clinic, Oral Medicine and Oral Surgery Department, Medical and Molecular Parasitology and Mycology Laboratory, LR12ES08, 5019 Monastir, Tunisia; ^3^University Dental Clinic, Pediatric Dentistry Department, Dento-Facial Biological and Clinical Approach Laboratory, LR12ES10, 5019 Monastir, Tunisia; ^4^University Dental Clinic, Outpatient Department, Research Laboratory of Oral Health and Orofacial Rehabilitation, LR12ES11 Monastir 5000, Tunisia

## Abstract

**Background:**

Mucocele is the most common minor salivary glands disease. Its management may present a challenge for dental professionals. The aim of the present clinical case was to describe mucocele treatment with diode laser and its benefits. *Case Report*. A case of lower lip mucocele in a 10-year-old female patient is reported. A conventional excision surgery was performed. Two months later, the patient reported discomfort and swelling at the same operative site. The lesion had recurred. Thus, mucocele was removed using a diode laser with wavelength of 980 nm, an initiated fiberoptic tip of 300 *μ*m, in continuous mode, and a power setting of 2 Watts. The procedure was rapidly completed with no bleeding. The patient was followed-up after 2 weeks and 6 months. The wound healed without complications: no postoperative discomfort or pain and no infection. There was no recurrence.

**Conclusion:**

Diode laser is an effective, easy, bloodless, and well-accepted procedure to treat mucocele in pediatric patients.

## 1. Introduction

Mucocele is a common oral lesion affecting minor salivary glands. It develops by extravasation or retention of mucous [1–4].

According to the literature, mucoceles occur more frequently in the lower lip [5–9]. This can be explained by the sharp borders of children' incisives biting lower lip, leading to a trauma or repeated stimulation [5, 10, 11].

The other affected sites are the tongue, palate, cheek, and floor of the mouth [9, 12]. Clinically, the lesion appears as a pink or bluish fluctuant nodule that may vary in color and size [5]. It may break off and resolve spontaneously [13, 14].

Many procedures were performed for mucocele removing: conventional surgery which is the most recommended method, electrosurgery, cryosurgery, micromarsupialization, and marsupialization. Steroids injection was also reported [2, 4, 8, 15, 16].

Many authors have described diode laser application for mucosal lesions in the oral cavity as an alternative to the precedent methods [17, 18].

Lasers offer many advantages such as ease of working, reduced healing time, and high affinity for melatonin and hemoglobin. Soft tissues may be cut, excised, or vaporized [18, 19].

The objective of this paper was to describe the use of diode laser to treat a recurrent mucocele in a young patient and its advantages.

## 2. Case Report

**Figure 1:**
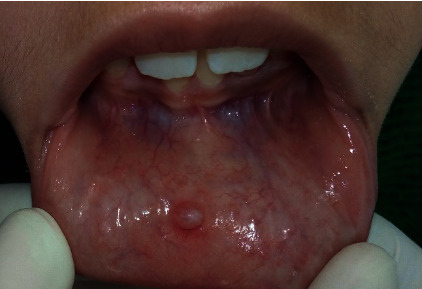
Clinical appearance of the mucocele in the lower lip.

A 10-year-old girl consulted the Pediatric Dentistry Department in Monastir University Dental Clinic, Tunisia. She complained about recurrent mucocele which was surgically removed in the last 2 months. The patient described discomfort and swelling in the right part of the lower lip. The lesion was increasing in size and symptoms appeared when biting her lip. History and clinical examination revealed a recurrent mucocele: a small pink nodule measuring 5 mm of diameter in the lower lip (Figure 1).

The treatment plan consisted on mucocele removal with laser application. Informed consent was obtained from the parent's patient. The procedure was conducted in accordance with the Helsinki Declaration.

Diode laser (Doctor Smile Simpler, Lambda, Italy) was used under local anesthesia (Medicaine 2% with adrenaline 1/100.000®, Medis, Tunisia).

This laser device emits photons at a wavelength of 980 nm and operates in a continuous emission mode with a supplementary gated emission. This device has a maximum power output of 8 W, with a repetition rate that can attain 25 kHz. The delivery system is a quartz fiber optic. For this specific case, a 300 *μ*m initiated tip was employed and the laser was set according to the following parameters:

- Peak power: 2 W
- Emission mode: continuous wave
- Average power: 2 W
- Length of treatment: 300 sec
- Tip-to-tissue distance: in contact
- Speed of movement: 1 mm/sec
- Total energy delivered: 600 joules

**Figure 2:**
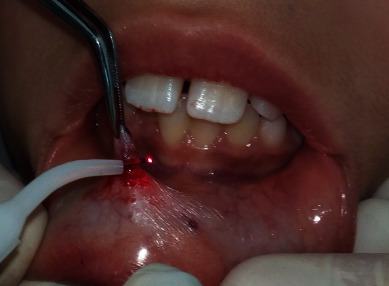
Diode laser tip handled around the lesion.

**Figure 3:**
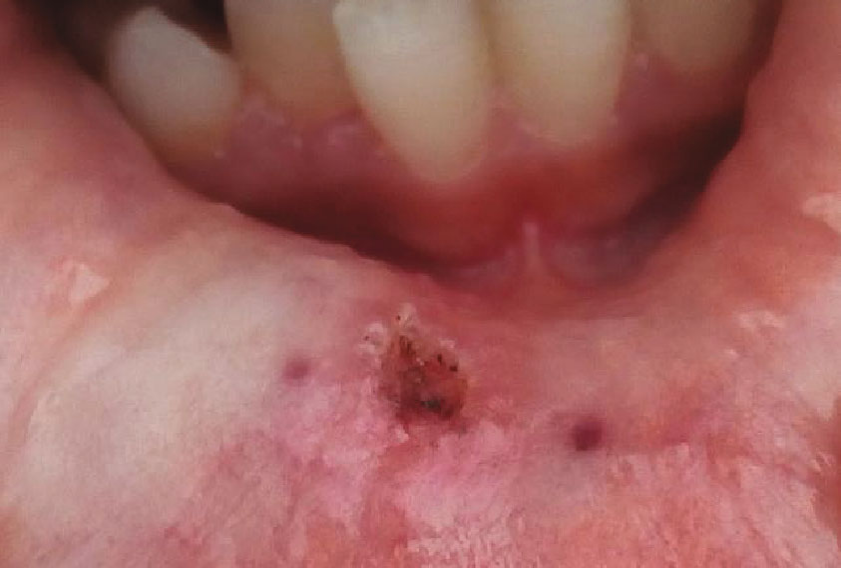
Immediate aspect after mucocele removal: bloodless operative site, no suture was done.

**Figure 4:**
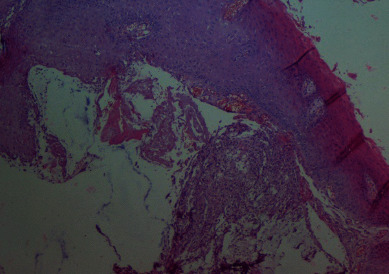
Histopathologic findings showing a regular Malpighian epithelium and a subepithelial connective tissue occupied by a cystic cavity which was surrounded by a granulation tissue (hematoxylin and eosin, original magnification, ×100).

All the practitioners and the patient wore laser eye protectors. The tip was directed to the surface of the lip at the base of the lesion at an angle of 10 to 15°. Movements were performed around the base, while the mucocele was grabbed by tweezers (Figure 2). The site was slowly and continuously mopped by sterile wet gauze to avoid tissues overheating. Care was taken also to always control the tip. If upon inspection, any damage or collection of debris was observed during treatment, the tip was cleaned with a sterile gauze. The mucocele was totally removed in 5 minutes. No bleeding was observed in the operative site and no sutures were necessary (Figure 3). The patient was told not to bite her lips and if healing was not complete by 4 weeks or any recurrence appeared, she should return for further examination and treatment. Histopathologic examination of removal tissue confirmed the diagnosis of mucocele. It showed a regular Malpighian epithelium, and a subepithelial connective tissue occupied by a cystic cavity which was surrounded by a granulation tissue. The infiltrate was rich in macrophages (Figure 4).

**Figure 5:**
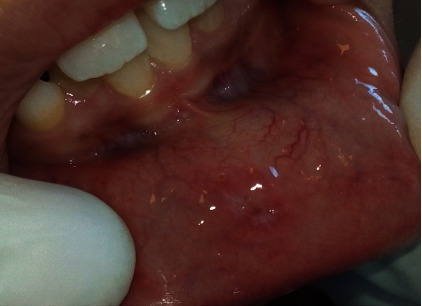
After 2 weeks of follow-up: fibrin network recovered the site.

**Figure 6:**
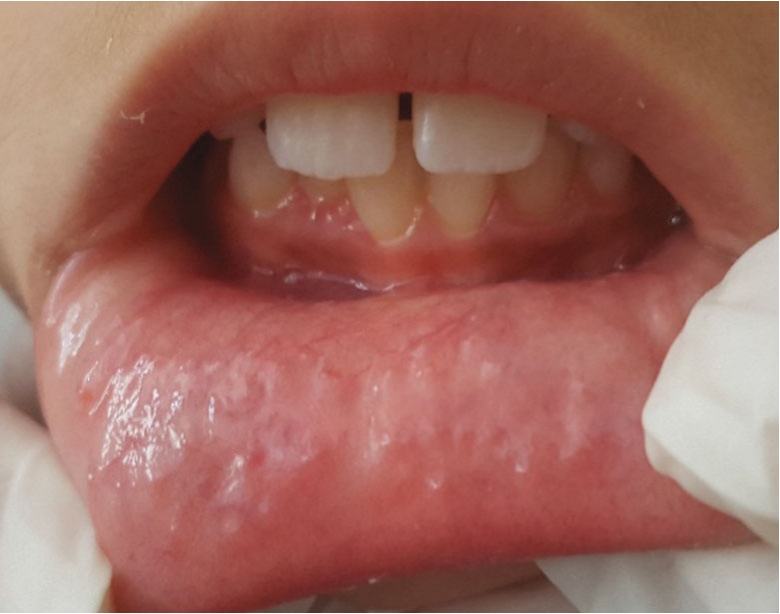
Perfect healing after 6 months of follow-up.

The child was followed after 2 weeks: a fibrin network formed over the surface (Figure 5). The wound healed without complications: no postoperative discomfort or pain and no infection were noted. No recurrence was observed. The surface of the lip healed perfectly after 6 months of follow-up (Figure 6).

## 3. Discussion

Mucoceles are frequent benign lesions in young individuals [8]. In the present case, a mucocele on a young girl was treated two times. At first time, a scalpel excision was done. After recurrence, we decided to use diode laser approach.

Etiologic factor of mucous cyst development in the lower lip may be caused by chronic trauma arising out of feeding and biting habit that can initiate inflammatory or hemorrhagic phenomena [20, 21]. Moreover, saliva secreted in the oral cavity by salivary glands through ducts. If these ducts are blocked or traumatized, the saliva is collected at the cut spot leading to swelling or a mucocele [2, 8, 9, 13, 22–24].

Surgical extirpation of mucoceles is the most common treatment [15]. However, the method itself can traumatize tissues and cause recurrence [25].

In this case, clinical features and history indicate the recurrence of the lesion: same location, history of trauma, and rapid appearance. Besides, the patient had a habit of biting her lower lip by the borders of the anterior maxillary teeth. The space between incisors and mechanical stimulation may be the cause of mucocele appearance and recurrence [10]. Therefore, the patient was encouraged to stop her bad habit.

The important practical points to be considered leading to a successful operation without recurrence are excising the cyst and the adjacent minor salivary glands and extending the removed area to the muscle layer [12, 19].

In the present case, the surgery was completed in 5 minutes. This characteristic is in accordance with other authors' observations [12]. It was also bloodless and there was no need for suture.

Laser diode treatment has proven a satisfying result when used to treat oral soft tissues [19] especially for young patients [7].

Our findings showed that this latter has many advantages: good hemostasis during and after operation, procedure speed which minimizes discomfort especially for children and lack of complications during or after application.

Laser diodes are used to manage oral soft tissues because they is highly absorbed by water and hemoglobin, melanin, and collagen chromophores and poorly absorbed by dental hard tissues [21, 26, 27]. Laser provides cut and coagulation at the mean time so bleeding is significantly reduced or even absent. There is no need to suture the operating site [17, 27, 28]. In addition, the site is immediately disinfected by the laser [17]. This tool enhances wound healing without infection or swelling because it has antibacterial and anti-inflammatory properties [21, 29]. These effects are mostly desired in developing countries who have higher postoperative complications [20, 21].

Tunisia has an arid climate with humidity zones. Besides, the child came from a rural region where the control infection measures may be unavailable. These conditions rise a concern about the possibility of secondary infection occurrence which prompt caution regarding contamination. But in this case, the need for medication was eliminated. The girl was confident and more cooperative when laser was used. Furthermore, she began to reduce her biting habit. Consequently, the healing was perfect without any scarring or infection and the patient did not complain about any unpleasant feeling.

Diode laser improve wound recovery. Cicatrization time is shorter than after conventional surgery [21]. Six months of follow-up did not show any recurrence.

When done with care, this alternative may be more successful and helpful with less recurrence, less postoperative discomfort for pediatric patients.

Usually the diode lasers are associated with aluminum, gallium, and arsenic [21]. They have a wavelength between 810 nm and 980 nm [18]. Laser irradiation for oral soft tissue procedures can be in continuous mode or pulsed mode [18]. It can be used for ablation, incision, excision coagulation, and hemostasis [18, 21].

It makes sense to know that this type of laser produces a rapid increase in the temperature of the target tissue [18]. Thus, particular attention must be paid to the time of application and the working power in order to prevent adjacent tissues overheating and necrosis [21].

This clinical case agrees with previous reports substantiating that diode laser treatment may be a good therapeutic alternative for oral lesions and particularly suitable for infants. However, more studies are needed to compare long-term efficacy of this device with other laser types.

## 4. Conclusion

Diode laser application is rapid, efficient, and safe. It is well-accepted by young patients because it is painless and has no postoperative complications. The comfort provided by this technique spurs dental practitioners to use it in their routine work.

## Data Availability

The data (figures) used to support the findings of this study are included within the article.
